# 

**Published:** 1858-11

**Authors:** 


NORTH AMERICAN

### MEDICO-CHIRURGICAL REVIEW.

Vol. II.] NOVEMBER, 1858. [No. 6.

### CONTENTS.

### ANALYTICAL AND CRITICAL REVIEWS.

ART. PAGE

I.—De la Prostitution dans la Ville de Paris, etc. Par A. J. B. Parent-Duchatelet. . . . . . . . . . 977

II.—An Essay on the Pathology and Therapeutics of Scarlet Fever. By Caspar Morris, M.D. . . . . . . . . . 1010

III.—The Uræmic Convulsions of Pregnancy, Parturition, and Child-bed. By Carl R. Braun, M.D. . . . . . . . 1020

IV.—On the Pathology, Symptoms, and Treatment of Ulcer of the Stomach; with an Appendix of Cases. By Wm. Brinton, M.D. . . . . . . . . . 1028

V.—Ventilation in American Dwellings; with a Series of Diagrams presenting Examples in Different Classes of Habitations. By David Boswell Reid, M.D., etc. . . . . . . 1035

VI.—A Treatise on the Practice of Medicine. By George B. Wood, M.D. . . . . . . . . . 1036

VII.—A Manual of Practical Medicine. By T. H. Tanner, M.D. . 1036

VIII.—The Physician's Visiting List, Diary, and Book of Engagements, for 1859. . . . . . . . . . . 1037

### ORIGINAL COMMUNICATIONS.

I.—Observations on the Deaf and Dumb. By Richard J. Dunglison, M.D. . . . . . . . . . . 1037

II.—Surgical Cases. By A. Lopez, M.D. . . . . . . . 1065

III.—Proceedings of the Pathological Society of Philadelphia. . . 1072

### AMERICAN PROGRESS OF MEDICAL SCIENCE.

Report on Materia Medica and Therapeutics. By John Bell, M.D. Part II. . . . . . . . . . . . . . . 1079

### BI-MONTHLY FOREIGN ABSTRACTS.

#### CHEMISTRY AND PHYSIOLOGY.

I.—Researches on the Chemical Composition of the Brain. By M. Müller. . . . . . . . . . . . . 1104

II.—On a New Basic Substance Extracted from Muscles. By A. Strocker. . . . . . . . . . . 1104

III.—Influence of the Spinal Marrow on the Brain. By Dr. Brown-Séquard. . . . . . . . . 1104

IV.—Measurement of the Sensibility in the Morbid State. By Dr. Brown-Séquard. . . . . . . . . . . 1105

V.—Serous Sac of the Middle Ventricle of the Brain. By Prof. Förster. 1105

ART. PAGE
VI.—Anæsthesia Universalis Peripherica. By Dr. BINZ. . . . . 1106
VII.—Pericarditis, with Acute Fatty Degeneration of the Heart. By Prof. VIRCHOW. . . . . . . . . . . . 1106
VIII.—Observations on Pulmonary Tuberculosis. By Prof. LEBERT. . 1107
IX.—1. Acute Yellow Atrophy of the Liver, with Icterus. By Dr. MUHLIG. . . . . . . . . . . . . 1108
2. Similar Case. By Prof. BAMBERGER. . . . . . . 1108
X.—On Nutmeg Liver. By Dr. ANT. REZZONICO. . . . . . 1110
XI.—Observations on Cholera. By Prof. LEBERT. . . . . . 1111
XII.—On Prolonged Constipation. By Prof. TEISSIER. Reported by Dr. COUTAGNE. . . . . . . . . . . 1113
XIII.—On the Observations of Temperature in Patients. By Prof. WUNDERLICH and Dr. MEYER. . . . . . . . . . 1114

MATERIA MEDICA.

XIV.—Argenti Nitras in Oxyuris Vermicularis. By Dr. SCHULTZ. . 1116
XV.—Iodide of Potassium as Antigalactic. By Prof. ROUSSET. . . 1116
XVI.—Cerate of Opium in Carbuncle. By Dr. VON GUTZEIT. . . 1117

### BI-MONTHLY PERISCOPE.

SURGERY.

1. Twelve Cases Illustrating the Treatment of Spina Bifida. . . . 1117
2. Account of some Trials made to facilitate the Removal of Stones from the Urinary Bladder. . . . . . . . . . 1127

PRACTICAL MEDICINE.

1. An Account of some Cases of Scarlatina Anginosa successfully treated by Hot Packing . . . . . . . . . . 1129
2. Treatment and Clinical History of Asthma. . . . . . . 1134

OBSTETRIC MEDICINE.

1. A New Symptom of Rupture of the Uterus. . . . . . . 1137
2. Puerperal Convulsions. . . . . . . . . . . 1138
3. Statistics of Operative Midwifery. . . . . . . . . 1142
4. Influence of Pregnancy and Delivery upon Insanity. . . . . 1145

EDITORS' TABLE.—Allopathy—Allopathists. . . . . . . . 1146
Craniography. . . . . . . . . . . . . . 1148
Appointments in Medical Schools. . . . . . . . . 1149
New York State Inebriate Asylum. . . . . . . . . 1149
Contributions to Obstetric Jurisprudence. . . . . . . . 1149
Correction. . . . . . . . . . . . . . 1150
The Medical and Surgical Reporter. . . . . . . . . 1150
Vesico-vaginal Fistule in Europe. . . . . . . . . 1150
Ecraseur. . . . . . . . . . . . . . . 1151
The Pathological Society of Philadelphia. . . . . . . 1152

NECROLOGICAL NOTICES.—Mr. George Combe . . . . . . . 1152
Dr. Edward Minturn. . . . . . . . . . . 1153

BI-MONTHLY BIBLIOGRAPHICAL RECORD . . . . . . . . 1154

Index to Volume II. . . . . . . . . . . . . 1155

